# Comparative Proteomic Analysis of Popcorn Genotypes Identifies Differentially Accumulated Proteins Associated with Resistance Pathways to Southern Leaf Blight Disease

**DOI:** 10.3390/plants14030426

**Published:** 2025-02-01

**Authors:** Caio Cézar Guedes Corrêa, Tatiana Santos Barroso, Lucas Rodrigues Xavier, Vitor Batista Pinto, Ricardo Souza Reis, Guilherme Ferreira Pena, Claudete Santa-Catarina, Marcelo Vivas, Antonio Teixeira do Amaral Júnior, Vanildo Silveira

**Affiliations:** 1 Laboratório de Biotecnologia, Centro de Biociências e Biotecnologia (CBB), Universidade Estadual do Norte Fluminense Darcy Ribeiro (UENF), Av. Alberto Lamego 2000, Campos dos Goytacazes 28013-602, RJ, Brazil; caiocagronomo@gmail.com (C.C.G.C.); rxlucas@outlook.com (L.R.X.); ricardoreisreis@gmail.com (R.S.R.); 2 Unidade de Biologia Integrativa, Setor de Genômica e Proteômica, Universidade Estadual do Norte Fluminense Darcy Ribeiro (UENF), Av. Alberto Lamego 2000, Campos dos Goytacazes 28013-602, RJ, Brazil; 3 Departamento de Biologia, Centro de Ciências Exatas, Naturais e da Saúde, Universidade Federal do Espírito Santo, Alto Universitário s/n, Alegre 29500-000, ES, Brazil; 4 Laboratório de Biologia Celular e Tecidual, Centro de Biociências e Biotecnologia (CBB), Universidade Estadual do Norte Fluminense Darcy Ribeiro (UENF), Av. Alberto Lamego 2000, Campos dos Goytacazes 28013-602, RJ, Brazil; vitorbp@uenf.br (V.B.P.); claudete@uenf.br (C.S.-C.); 5 Laboratório de Melhoramento Genético Vegetal, Centro de Ciências e Tecnologias Agropecuárias (CCTA), Universidade Estadual do Norte Fluminense Darcy Ribeiro (UENF), Campos dos Goytacazes 28013-602, RJ, Brazil; penabio2@gmail.com (G.F.P.); amaraljr@uenf.br (A.T.d.A.J.); 6 Laboratório de Engenharia Agrícola, Centro de Ciências e Tecnologias Agropecuárias (CCTA), Universidade Estadual do Norte Fluminense Darcy Ribeiro (UENF), Campos dos Goytacazes 28013-602, RJ, Brazil; vivas@uenf.br

**Keywords:** biotic stress, comparative proteomics, fungus–plant interaction, southern leaf blight, *Zea mays*

## Abstract

Southern leaf blight (SLB), caused by *Bipolaris maydis*, poses a significant threat to maize and popcorn production. To understand the molecular mechanisms underlying SLB resistance, we conducted a high-throughput proteomic analysis comparing SLB-resistant (L66) and SLB-susceptible (L51) popcorn genotypes at four and ten days after inoculation (DAI). A total of 717 proteins were identified, with 151 differentially accumulated proteins (DAPs) between the genotypes. Eighteen DAPs exhibited the same regulatory pattern in both the SLB-resistant and SLB-susceptible genotypes at four (R4/S4) and ten (R10/S10) DAI. The protein-protein interaction (PPI) network of differentially accumulated proteins (DAPs) linked to SLB resistance and susceptibility enriched specific metabolic pathways in the SLB response, including photosynthesis, ribosome, ascorbate and aldarate metabolism, glutathione metabolism, and carbon metabolism. Proteins such as photosystem II 11 kD protein (B4FRJ4, PSB27-1), which was up-regulated at both time points (R4/S4 and R10/S10), and 60S acidic ribosomal protein P0 (A0A1D6LEZ7, RPP0B), which was unique to the resistant genotype at both time points (R4 and R10), highlighted the importance of maintaining photosynthetic efficiency and protein synthesis during pathogen attack. Additionally, dehydroascorbate reductase like-3 (B4F817, DHAR3) was consistently up-regulated at both time points in resistant genotypes, emphasizing its role in redox balance and ROS detoxification. In contrast, glyceraldehyde-3-phosphate dehydrogenase (K7UGF5, GAPC2), a glycolytic enzyme, was unique to the susceptible genotype, suggesting its involvement in managing energy metabolism under stress conditions. Our findings suggest that resistance to SLB in popcorn involves a combination of enhanced photosynthetic repair, redox homeostasis, and ribosomal protein activity, providing new potential molecular targets, such as DHAR3 and RPP0B, for genetic improvement in SLB resistance. These results offer valuable insights into breeding programs aimed at developing SLB-resistant popcorn varieties.

## 1. Introduction

Southern leaf blight (SLB) caused by *Bipolaris maydis* is a major threat to maize production worldwide. The disease is characterized by brown lesions on leaves, with a purplish tinge or reddish-brown margin, sometimes zonated, and elongated longitudinally [1]. Controlling SLB is challenging because of the ability of the pathogen to survive in agricultural residues and reinoculate subsequent crops, thus completing its life cycle [2]. Resistant genotypes offer the best control, but there are no SLB-resistant maize or popcorn cultivars available for commercial cultivation.

Host plants have evolved various defense mechanisms to avoid or reduce pathogen infection. They possess a surveillance system to detect invading pathogenic microorganisms and activate various defense mechanisms, including oxidative detoxification, antimicrobial compound production, callose deposition, and signaling pathways, leading to the presence of pathogen-related (PR) proteins [3,4].

Plants detect pathogens using two primary recognition systems: pathogen-associated molecular pattern (PAMP)-triggered immunity, which involves the recognition of conserved pathogen molecules by plant pattern recognition receptors (PRRs), and effector-triggered immunity (ETI) [5]. ETI relies on gene-for-gene interactions (PR-to-virulence) to neutralize specific pathogen effectors produced by various plant pathogens [3,5,6]. In this context, the use of tools such as metabolomics, proteomics, and transcriptomics is strategic in research aimed at understanding the molecular factors associated with modulating plant–pathogen interactions [7]. Thus, infection by pathogens causes an oxidative wave in plant cells that activates the post-translational modification of various proteins important for plant resistance, highlighting the role of proteomics in biotic stress studies [8].

Systemic acquired resistance (SAR) in plants involves the induction of proteins responsible for localized resistance reactions, called hypersensitive responses (HRs), and the accumulation of pathogenesis-related proteins, resulting in broad-spectrum systemic resistance [6,9,10]. The production of reactive oxygen species (ROS) is one of the first triggers for SAR activation, initiating a wave of signaling throughout the plant body which is essential for plant acclimation and defense against biotic and abiotic stresses [11]. Typically, SARs trigger prompt cell death at the infection site, facilitated by the production of ROS. In this context, the production and accumulation of ROS can also be a strategy used by pathogens to induce cell death and to feed on the nutrients that will be released, illustrating how important ROS homeostasis is during biotic stress [12].

Understanding the resistance mechanisms of popcorn against *B. maydis* is crucial for plant breeding to increase disease resistance. The use of omics-based systems of biology technology not only enhances our understanding of plant physiological processes and their regulatory and metabolic pathways but also aids in the identification of genes related to resistance [13]. The proteomics approach is a useful tool for identifying hotspots of biomarker candidates and exploring stress-responsive proteins or post-transcriptional modification sites to identify resistant plant varieties [14]. In recent study, comparative proteomic analyses revealed differentially abundant proteins that play key roles in modulating resistance against SLB in *Zea mays* [15].

In this study, we investigated differentially accumulated proteins (DAPs) associated with the defense response against *B. maydis* in two contrasting popcorn genotypes. We identified new players regulating the resistance mechanism against this pathogen and present the first high-throughput proteomic analysis of popcorn plants infected with *B. maydis*. Our findings expand the body of knowledge for further research on popcorn breeding.

## 2. Results

**Figure 1:**
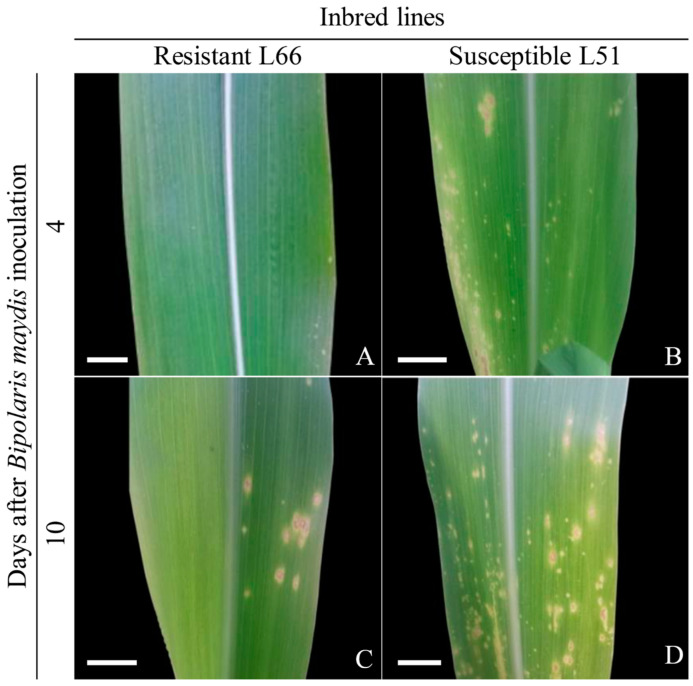
Popcorn leaves from the SLB-resistant genotype (L66) and the SLB-susceptible genotype (L51) at four and ten days after inoculation (DAI) with *Bipolaris maydis.* (**A**) L66 leaves at 4 DAI, showing no visible symptoms (scale 0). (**B**) L51 leaves at 4 DAI, displaying a moderate number of lesions on the lower leaves (scale 2). (**C**) L66 leaves at 10 DAI, exhibiting a few scattered lesions on the lower leaves (scale 1). (**D**) L51 leaves at 10 DAI, with abundant lesions spreading from the lower to the middle and upper leaves (scale 4).

The leaf lesions of the SLB-resistant (L66) and -susceptible (L51) plants exhibited distinct changes in response to infection by *B. maydis* (Figure 1). Considering the 0–5 scale of disease severity [16], the SLB-resistant plants presented minor lesions on the fourth and tenth DAI, rated as scale 0 (no disease) and scale 1 (one or two to few scattered lesions on the lower leaves), respectively (Figure 1A,C). In contrast, the SLB-susceptible plants had lesions rated as scale 2 (moderate number of lesions on lower leaves only) on the fourth day and scale 4 (lesions abundant on lower leaves and middle leaves, extending to upper leaves) on the tenth day (Figure 1B,D).

**Figure 2:**
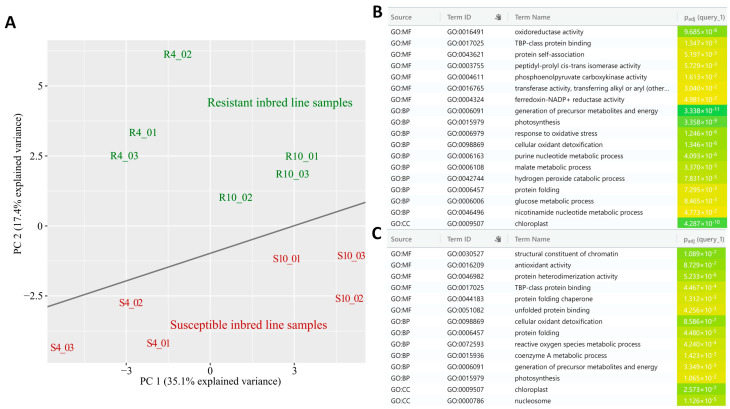
Data analysis for protein profiles of the SLB-resistant (L66) and SLB-susceptible (L51) genotypes at four and ten DAI. In the PCA (**A**), the triplicates of SLB-resistant plants (L66) at four and ten DAI were grouped together and are highlighted in green, whereas the triplicates of SLB-susceptible plants (L51) at four and ten DAI were grouped together and are highlighted in red. The enrichment analysis of biological process (BP), molecular function (MF), and cellular component (CC) terms was performed using the DAPs between R4/S4 (**B**) and R10/S10 (**C**).

In this study, we identified a total of 717 proteins (Supplementary Table S1), with 151 differentially abundant proteins (DAPs) identified in at least one of the comparisons performed. The PCA revealed a clear separation between the SLB-resistant (R4 and R10) and SLB-susceptible (S4 and S10) genotypes (Figure 2). PC1, which explains 35.1% of the variance, distinguishes the susceptible samples (S4 and S10) on the left from the resistant samples (R4 and R10) on the right. Although the complexity of proteomic data limits the cumulative variance, the PCA effectively highlights the distinction between resistant and susceptible genotypes. The tight clustering of biological replicates reflects consistency within the groups, highlighting distinct proteomic responses on the basis of genotype and time post-inoculation with *B. maydis*. In addition, the analysis of biological processes, cellular components, molecular functions, and metabolic pathways revealed that the resistant and susceptible genotypes regulated different groups of proteins after 4 (Figure 2B) and 10 days (Figure 2C) of infection. In this context, DAPs are mostly associated with photosynthesis, the response to oxidative stress and energy metabolism (Figure 2).

**Figure 3:**
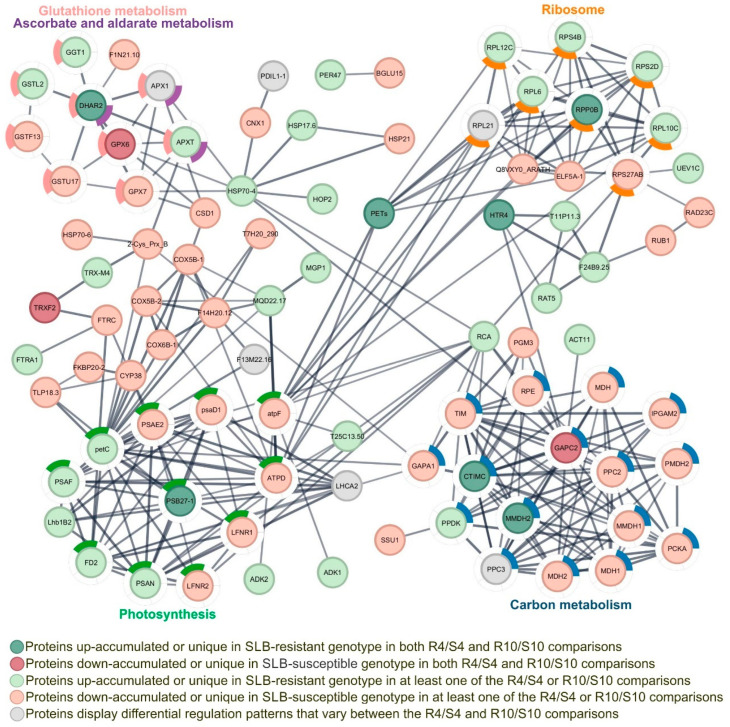
Protein-protein interaction (PPI) network of differentially accumulated proteins (DAPs) associated with SLB resistance and susceptibility. The PPI network was constructed on basis of KEGG-enriched metabolic pathways via the STRING database and visualized with Cytoscape. The key enriched pathways included photosynthesis, ribosome, ascorbate and aldarate metabolism, glutathione metabolism, and carbon metabolism. Node colors represent different regulatory patterns as indicated in the figure.

A comparison of the genotypes at four DAI (R4/S4) revealed 24 up-accumulated proteins, 20 unique to R4, 46 down-accumulated proteins, and 10 unique to S4 (Supplementary Table S1). In the R10/S10 comparison, 13 proteins were up-accumulated, 19 were unique to R10, 32 were down-accumulated, and 11 were unique to S10 (Supplementary Table S1). Eighteen DAPs were identified, exhibiting the same regulatory pattern in both the SLB-resistant and SLB-susceptible genotypes at four (R4/S4) and ten (R10/S10) DAI (Table 1). Among these DAPs, seven were enriched in the protein-protein interaction (PPI) network, which was constructed on the basis of KEGG-enriched metabolic pathways. These proteins were associated with specific enriched pathways, including photosynthesis (B4FRJ4, PSB27-1), ribosome (A0A1D6LEZ7, RPP0B), glutathione metabolism (B4F817, DHAR3), ascorbate and aldarate metabolism (B4F817, DHAR3; B4FRF0, GPX6), and carbon metabolism (K7UGF5, GAPC2; A0A1D6ETY3, CTIMC; A0A1D6FKV9, MMDH2) (Figure 3; Table 1). The complete list of KEGG gene ontology and metabolic pathways is available in Supplementary Table S2 (DAPs from time 4) and Supplementary Table S3 (DAPs from time 10).

In the photosynthesis pathway, five proteins were more abundant in the SLB-resistant genotype at least one time point, including B4FRJ4 (PSB27-1), a photosystem II 11 kD protein, which was up-regulated in both comparisons (R4/S4 and R10/S10), and six proteins were more abundant in the SLB-susceptible genotype (Figure 3; Supplementary Table S1).

In the ribosome pathway, six proteins were more abundant in the SLB-resistant genotype, including A0A1D6LEZ7 (RPP0B), a 60S acidic ribosomal protein P0, which was unique to the resistant genotype at both time points (R4 and R10) (Figure 3; Supplementary Table S1). In contrast, the protein P27923 (RPS27AB) was more abundant in the susceptible genotype in the R10/S10 comparison (Figure 3; Supplementary Table S1).

In the ascorbate and aldarate metabolism pathway, two proteins were more abundant in the resistant genotype, including B4F817 (DHAR3), a dehydroascorbate reductase like-3, which was up-regulated in both comparisons, while one protein was more abundant in the susceptible genotype (Figure 3; Supplementary Table S1). In the glutathione metabolism pathway, four proteins were more abundant in the resistant genotype, including a DHAR3, while four proteins were more abundant in the susceptible genotype, including B4FRF0 (GPX6), a glutathione peroxidase, which was unique in both S4 and S10 time points (Figure 3; Supplementary Table S1).

Finally, in the carbon metabolism pathway, twelve proteins were more abundant in the susceptible genotype, including K7UGF5 (GAPC2), a glyceraldehyde-3-phosphate dehydrogenase, which was unique to the susceptible genotype at both time points (S4 and S10) (Figure 3; Supplementary Table S1), whereas three proteins were more abundant in the resistant genotype (Figure 3; Supplementary Table S1).

## 3. Discussion

The observed resistance of the L66 genotype to *B. maydis* highlights its potential as a source of valuable traits for breeding programs aimed at improving SLB resistance. The limited lesion development in resistant plants suggests the activation of defense mechanisms early in the infection process, likely preventing the spread of the pathogen. Given the complexity of plant–fungus interactions, further understanding of these mechanisms is essential. In this study, we employed high-throughput proteomics to identify potential new molecular players involved in the resistance response. Fungal pathogens remain one of the most significant challenges in crop production, and while certain resistance pathways have been elucidated, how plants such as popcorn establish effective defenses is still unclear.

Our proteomic analysis revealed a wide range of DAPs in the SLB-contrasting popcorn genotypes after *B. maydis* inoculation. The analysis of DAPs in the context of SLB resistance in maize revealed the involvement of specific metabolic pathways that are enriched in the PPI network. These pathways include photosynthesis, ribosome assembly, ascorbate and aldarate metabolism, glutathione metabolism, and carbon metabolism. These enriched pathways seem to be candidates that play important roles in modulating the plant’s response to pathogen stress, contributing to either increased resistance or increased susceptibility. Although no proteins induced by SAR were identified as DAPs, four members of the pathogenesis-related family (B4FVP5, A0A1D6HRU2, Q29SB6, and K7UPV6; Supplementary Table S1) were identified in our dataset; however, none of them met the statistical criteria to be classified as differentially regulated, highlighting the complexity of defense-related protein regulation in this context. Nevertheless, our data suggest that resistance to infection is associated with better ROS homeostasis in the resistant genotype, which is an important factor for the induction of SAR during biotic stress [11,12]. ROS production is fundamental for perception, cell signaling, and response to pathogen attack, but excessive accumulation of these molecules can induce oxidative stress and cell death, where ROS-detoxifying enzymes play essential roles during infection [11].

In the context of plant responses to biotic stress, photosynthesis-related genes are commonly observed to be globally down-regulated during abiotic and biotic stress [17]. This down-regulation serves as part of the plant’s strategy to divert energy and resources away from growth-related processes, such as photosynthesis, which allows the plant to focus on mitigating pathogen damage. However, in contrast to these findings, our study reveals a different regulatory pattern in the SLB-resistant genotype. In our work, five proteins revealed to photosynthesis were more abundant in the SLB-resistant genotype, including a photosystem II 11 kD protein (B4FRJ4, PSB27-1), which was up-regulated at both time points (R4/S4 and R10/S10). The photosystem II protein is crucial for the assembly and repair of photosystem II, and its up-regulation in resistant genotypes suggests that maintaining the integrity and functionality of PSII is a key component of the plant’s defense strategy against SLB.

The up-regulation of the photosystem II protein in resistant genotypes may reflect a strategy to preserve photosynthetic efficiency and avoid the suppression of critical photosynthetic processes during pathogen attack. This could allow the plant to balance energy production while simultaneously activating defense responses. Photosystem II proteins, such as PSB27, are involved in repair mechanisms that mitigate damage caused by ROS, which are typically overproduced during both biotic and abiotic stress [18]. By maintaining photosystem II functionality, plants can reduce ROS buildup and prevent oxidative damage to the photosynthetic machinery, which might explain the enhanced resistance observed in the SLB-resistant genotype in our study. The interplay between glutathione metabolism and photosynthesis may further support our findings since glutathione plays a role in regulating redox homeostasis and the detoxification of ROS [19]. The increased abundance of proteins involved in the ascorbate–glutathione cycle (such as dehydroascorbate reductase like-3—DHAR3) indicates that SLB-resistant plants likely utilize a dual strategy of photosystem protection and redox balance to counteract pathogen-induced oxidative stress. This combination of enhanced photosynthetic repair and antioxidant defense mechanisms allows for more efficient handling of biotic stress.

In the glutathione metabolism pathway, in addition to DHAR3 being more abundant in the SLB-resistant genotype, the glutathione peroxidase B4FRF0 (GPX6) was uniquely accumulated in the SLB-susceptible genotype. DHARs and GPXs play crucial roles in managing oxidative stress, but they operate through different mechanisms, suggesting that the genotypes may have distinct strategies for handling stress. DHAR participates in this cycle, regenerating ascorbate from dehydroascorbate using glutathione as a reducing agent [20]. Together, APXs and DHAR enzymes work to maintain redox homeostasis within plant cells, particularly in chloroplasts and mitochondria [20]. DHAR proteins are also known to play a role in scavenging ROS as part of the plant’s antioxidant defense mechanisms [20]. One of the primary systems for hydrogen peroxide detoxification in plant cells is the ascorbate–glutathione cycle. Within this cycle, ascorbate peroxidase (APX) enzymes play a crucial role by catalyzing the reduction of hydrogen peroxide (H_2_O_2_) to water (H_2_O), utilizing ascorbate as a specific electron donor [21]. These proteins must be important for maintaining homeostasis during the signaling exerted by the ROS wave triggered by infection [11,12].

Numerous GPXs across different plant species have demonstrated their ability to protect cells from oxidative damage, particularly under stress conditions, by acting as potent ROS scavengers [22]. GPXs are essential antioxidant enzymes that play pivotal roles in managing ROS by catalyzing the conversion of H_2_O_2_ and other organic hydroperoxides into water or alcohols to maintain H_2_O_2_ homeostasis [23]. In our study, the greater abundance of GPX6 in the SLB-susceptible genotype suggests that the plants may have experienced elevated levels of ROS due to pathogen infection, triggering the synthesis of GPX6 as a reactive antioxidant defense. However, this response might not be sufficient to fully counter the oxidative damage, which could explain the increased susceptibility observed in these plants. The GPX pathway, while critical for detoxification, may not be as effective as the DHAR3-driven redox cycling observed in the SLB-resistant genotype, which maintains a more balanced oxidative state.

The DHAR3 protein plays a central role in two enriched metabolic pathways: ascorbate and aldarate metabolism and glutathione metabolism. Its consistent up-regulation in both comparisons (R4/S4 and R10/S10) across these pathways underscores its central function in managing redox balance and enhancing the plant’s defense against pathogen-induced oxidative stress. In a previous proteomic study in maize, ascorbate peroxidase 1 (ZmAPX1), encoded by a gene located within a quantitative trait locus associated with SLB resistance, was identified as a potential gene for increasing SLB resistance [15]. In our work with popcorn, we observed seven ascorbate peroxidases (A0A1D6ESK0, A0A1D6EAC4, A0A1D6JYW7, B6TM55, A0A1D6HAE3, A0A1D6QMU5, and B4G031), with a chloroplastic L-ascorbate peroxidase (A0A1D6EAC4, APXT) up-regulated in the R4/S4 comparison and unchanged in the R10/S10 comparison, whereas a cytosolic L-ascorbate peroxidase (A0A1D6ESK0, APX1) was up-regulated in R4/S4 and down-regulated in R10/S10.

Pathogens often employ strategies to deactivate certain signaling-related genes upon plant–pathogen interactions [24]. The regulation of ROS accumulation through the activity of enzymes such as DHARs and L-ascorbate peroxidases may represent an important defense mechanism in maize, supporting the hypothesis that redox balance proteins are central to the defense strategy against SLB [15]. Just as ZmAPX1 was identified as a potential target for improving resistance to SLB in maize [15], DHAR3 may also represent a promising candidate for genetic transformation or genome editing aimed at enhancing resistance to SLB, providing a dual approach to increase antioxidant defenses.

The involvement of ribosomal proteins in plant defense against pathogen-induced stress is an emerging field of study [25]. Although the protein translation machinery is fundamental to the growth and development of plants, its specific role in biotic stress tolerance has been less extensively explored. Recent studies demonstrated that ribosomal protein (RP) mutations and deficiencies can significantly influence a plant’s ability to resist diseases [25,26,27,28]. For instance, silencing RPL12 and RPL19 in both *Nicotiana benthamiana* and *Arabidopsis thaliana* impaired nonhost disease resistance against multiple bacterial pathogens [26]. Similarly, suppressing RPL10 reduced resistance to the nonhost pathogen *Pseudomonas syringae* pv. tomato T1 in both species [28]. Additionally, silencing RPS6 in *N. benthamiana* impacted the accumulation of various viruses, including cucumber mosaic virus, Turnip mosaic virus (TuMV), and potato virus A (PVA) [27].

In our study, we also observed that several ribosomal proteins were up-accumulated or unique to the SLB-resistant genotype, which aligns with these previous findings. Specifically, RPL10C, RPL12C, RPL6, RPS2D, and RPS4B were more abundant in the resistant genotype in at least one of the R4/S4 or R10/S10 comparisons, indicating their potential involvement in enhancing resistance to popcorn SLB. Moreover, RPP0B, a 60S acidic ribosomal protein P0, was unique to the resistant genotype at both time points (R4 and R10), further supporting the hypothesis that ribosomal proteins play a critical role in plant immunity by regulating the translation of defense-related proteins. These findings indicate that ribosomal proteins not only contribute to normal cellular functions but also play pivotal roles in plant immune responses by regulating the synthesis of defense-related proteins. Therefore, the modulation of ribosomal protein activity, such as the up-regulation of RPP0B (60S acidic ribosomal protein P0) in the SLB-resistant popcorn genotype, may be a critical factor in enhancing tolerance to pathogen-induced stress in plants.

The role of RPP0s proteins in plant immunity is highlighted through their involvement in ribosomal P-stalk regulation during pathogen-induced stress [29]. P-stalk, which includes RPP0s and other acidic ribosomal proteins, such as RPP1s and RPP2s, plays a significant role in the translocation step during protein translation. This translocation is crucial for elongation factor activity and protein synthesis. Importantly, mutants deficient in RPP0C, a homolog of RPP0B, were shown to be more susceptible to infection by *Pseudomonas syringae*, providing strong evidence that RPP0C plays a key role in plant immunity [29]. The importance of P-stalk, including RPP0B, in ribosomal reprogramming during stress underscores its role in ensuring efficient translation of defense proteins, highlighting RPP0B as a critical factor in plant pathogen defense.

In the carbon metabolism pathway, 12 proteins were more abundant in the SLB-susceptible genotype, including K7UGF5 (GAPC2), a cytosolic glyceraldehyde-3-phosphate dehydrogenase (GAPC2), which was uniquely expressed in the susceptible genotype at both time points (S4 and S10). GAPC2 is an important enzyme in glycolysis, known for catalyzing the initial step of the pathway by converting D-glyceraldehyde 3-phosphate (G3P) into 3-phospho-D-glyceroyl phosphate. This reaction is essential for sustaining cellular ATP levels and facilitating carbohydrate metabolism. In plants, GAPC2 has been shown to be involved in redox regulation and energy metabolism, particularly in response to various stress conditions such as salt stress and oxidative stress, which can affect other metabolic processes beyond glycolysis [30,31]. In addition, this enzyme is part of a regulatory mechanism in leaves that integrates starch metabolism with cell growth and expansion modulated by FERONIA, a protein kinase involved in hormonal responses and ROS production [32,33]. The complex interplay between GAPC1 and the cellular redox environment suggests its function as an oxidative stress sensor [31]. In this context, it is already known that infection by fungi and stimuli using elicitors such as salicylic acid are factors that induce the expression of GAPC in leaves and branches of *Solanum tuberosum* L., highlighting its role in responses to biotic stress [34]. New research into the enzymatic activity of GAPC and the accumulation of ROS during the infection of maize plants by fungi may provide new clues about the role of this enzyme in plant resistance to pathogen attack.

Other proteins in the carbon metabolism pathway, such as malate dehydrogenase (MDH) and phosphoenolpyruvate carboxylase (PCKA), that play pivotal roles in the C4 photosynthetic pathway, further support the idea that carbon metabolism is heavily impacted during SLB infection, and the differential expression of these enzymes in resistant and susceptible genotypes highlights the significance of this metabolic pathway in response to biotic stress. In photosynthetic tissues, reduced MDH activity impairs growth due to changes in photorespiration and CO_2_ assimilation rates in *Arabidopsis* [35]. The MDH enzyme is also important for modulating the energy balance and plant responses to different stresses [36]. In the roots, NaCl stress led to the differential regulation of genes and proteins involved in key respiratory pathways, such as glycolysis, the tricarboxylic acid cycle, and the pentose phosphate pathway [37,38]. The PCKA protein is known to accumulate mainly in the leaves and participate in carbon fixation, as well as improving plant resistance to abiotic stresses such as salinity and drought [39,40]. This suggests the involvement of these pathways in the plant’s response to biotic and abiotic stresses.

In our study, the down-regulation of proteins in the carbon metabolism pathway in the R4/S4 and/or R10/S10 comparisons, along with GAPC2 being unique to the SLB-susceptible genotype, suggests that this differential accumulation may be part of the plant’s attempt to regulate energy metabolism and maintain redox balance in response to stress induced by SLB infection.

## 4. Materials and Methods

### 4.1. Plant Material

The Popcorn Breeding Program at the UENF in Campos dos Goytacazes, Rio de Janeiro, Brazil, developed two inbred lines of popcorn (*Zea mays* var. everta) with contrasting responses to the *B. maydis* fungus. Among 37 inbred lines screened for genetic resistance to *B. maydis*-induced fungal leaf diseases, the L66 genotype was found to be resistant, whereas the L51 genotype was susceptible to the pathogen [41]. These two genotypes were used in this work.

### 4.2. Fungal Isolates

In this study, the *B. maydis* isolate CF/UENF 501, maintained at the UENF Phytosanitary Clinic, was used. The isolate was obtained from popcorn leaves displaying typical symptoms of *B. maydis* infection during the spontaneous emergence of the disease in the germplasm multiplication fields of the Popcorn Breeding Program at UENF. The reproductive structures of the fungus were observed under an Eclipse 80i optical microscope (Nikon, Tokyo, Japan), revealing curved conidia tapered at the ends with 3 to 13 septa and bipolar germination. The isolates were cultured on Petri plate dishes containing PDA culture medium (P2182; Merck Millipore, Burlington, MA, USA) and incubated for ten days at 25 °C with a 12 h photoperiod in a growth incubator (Eletrolab, São Paulo, SP, Brazil).

### 4.3. Plant Growth and Inoculation

To carry out the experiment, black plastic pots with a capacity of 5 L were filled with a mixture of washed sand and organic plant substrate (Bioplant, Nova Ponte, MG, Brazil) at a ratio of 1:1 (*v*/*v*) and kept in a greenhouse. The seeds of the different genotypes were sown in pots, and the plants were watered daily. Each pot contained three plants, and five pots were used for each experimental condition. A completely randomized design was used for the experiment.

At the V4 growth stage, the plants were inoculated with *B. maydis* by spraying a conidial suspension (2 × 104 conidia mL^−1^) on the leaves until the point of drainage. The plants were evaluated for morphological characteristics related to leaf lesions at four and ten DAI. The leaf lesions were recorded on a 0–5 scale of disease severity used in previous studies on maize infection by *B. maydis*, according to Hussain et al. [16].

### 4.4. Protein Extraction

Protein extracts were prepared using three biological replicates of leaves collected from plants at the V4 growth stage, sampled four and ten DAI. Each biological replicate consisted of pooled leaves from three popcorn plants. Proteins were extracted from 300 mg of fresh tissue using a modified trichloroacetic acid/acetone protocol [42]. Initially, the samples were suspended in a cold buffer containing 10% (*w*/*v*) trichloroacetic acid (Sigma-Aldrich, St. Louis, MO, USA) in acetone (Merck, Darmstadt, Germany) with 20 mM dithiothreitol (DTT; Bio-Rad Laboratories, Hercules, CA, USA), then vortexed for 5 min at 8 °C. The mixture was incubated at −20 °C for 1 h and centrifuged at 16,000× *g* for 30 min at 4 °C. Pellets were washed three times with cold acetone containing 20 mM DTT, air-dried, and resuspended in a buffer composed of 7 M urea (Cytiva, Marlborough, MA, USA), 2 M thiourea (Cytiva), 2% Triton X-100 (Sigma-Aldrich), 1% DTT, 1 mM PMSF (Sigma-Aldrich), and a complete protease inhibitor cocktail (Roche Diagnostics, Mannheim, Germany). Samples were vortexed for 30 min at 8 °C, followed by centrifugation at 16,000× *g* for 20 min. The resulting supernatants were collected, and protein concentrations were measured using a 2-D Quant Kit (Cytiva).

### 4.5. Protein Digestion

To prepare for protein digestion, 100 µg aliquots of each sample were processed. Initially, proteins were precipitated using the methanol/chloroform method to eliminate detergent and contaminants [43]. The samples were then resuspended in a buffer containing 7 M urea and 2 M thiourea, desalted using Amicon Ultra-0.5 3 kDa centrifugal filters (Merck Millipore), and digested following the protocol described by Passamani et al. [44]. Briefly, 25 μL of 0.2% (*v*/*v*) RapiGest^®^ (Waters, Milford, MA, USA) was added to each sample, vortexed, and incubated at 80 °C for 15 min. Subsequently, 2.5 μL of 100 mM DTT was added, and the samples were incubated at 60 °C for 30 min with constant agitation. Next, 2.5 μL of 300 mM iodoacetamide (Cytiva) was added, vortexed, and incubated in the dark for 30 min. To neutralize excess iodoacetamide, 5 µL of 100 mM DTT was added. Protein digestion was carried out by adding 20 μL of trypsin solution (50 ng.μL^−1^; V5111, Promega, Madison, WI, USA), followed by incubation at 37 °C for 15 h. To precipitate RapiGest^®^ and inhibit trypsin activity, 10 μL of 5% (*v*/*v*) trifluoroacetic acid was added, and the samples were incubated at 37 °C for 30 min. The mixture was centrifuged at 16,000× *g* for 20 min, and the resulting supernatants were transferred to total recovery vials (Waters).

### 4.6. Mass Spectrometry Analysis

To analyze the protein samples, nano-LC-ESI-MS/MS was used (nanoAcquity UPLC coupled to a Synapt G2-Si mass spectrometer; Waters) according to Passamani et al. [44]. The process began with the normalization of the relative protein concentrations by loading 1 µg of the digested samples. Normalization among samples was based on stoichiometric measurements of the sample total ion counts (TIC) of MS^E^ scouting runs prior to the analyses using the ProteinLynx Global SERVER software v.3.02 (PLGS; Waters).

After the normalization of the injection volumes, the peptide mixtures (2 µg) were separated onto a nanoAcquity UPLC 5 μm C18 trap column (180 μm × 20 mm; Waters) followed by loading onto a nanoAcquity HSS T3 1.8 μm analytical column (75 μm × 150 mm; Waters) at a rate of 400 nL min^−1^ and a temperature of 45 °C. Mobile phase A consisted of MS water (Tedia, Fairfield, OH, USA) and 0.1% formic acid (Sigma-Aldrich), while mobile phase B consisted of acetonitrile (Merck Millipore) and 0.1% formic acid. The gradient elution started at 7% of solution B and increased to 40% of solution B for 91.12 min.

MS analysis was performed both in positive and resolution modes with a full width at half maximum value of 35,000 and ion mobility and in data-independent acquisition mode (HDMS^E^). The ion mobility separation used a wave velocity of 600 m s^−1^; the transfer collision energy increased from 19 V to 55 V in high-energy mode; the cone and capillary voltages were 30 V and 2750 V, respectively; and the source temperature was 70 °C. For the TOF parameters, the scan time was set to 0.5 s in continuum mode, and the mass range was 50–2000 Da. An external calibrant, human [Glu1] fibrinopeptide B, at 100 fmol was used, and lock mass acquisition was performed every 30 s. MassLynx v.4.1 software (Waters) was used for mass spectrum acquisition.

### 4.7. Protein Identification and Functional Analysis

Spectral processing and database searching were performed using the PLGS software according to Passamani et al. [44]. The HDMS^E^ analysis parameters included a low-energy threshold of 150 counts, an elevated-energy threshold of 50 counts, an intensity threshold of 750 counts, one missed cleavage, a minimum of three fragment ions per peptide, a minimum of seven fragment ions per protein, a minimum of two peptides per protein, and false discovery rate (FDR) < 1%. Carbamidomethyl (C) was fixed as a modification, while oxidation (M) and phosphoryl (STY) were variable modifications.

Protein identification was conducted using the reference proteome for *Z. mays* L. databank from UniProtKB (Proteome ID: UP000007305). The label-free quantification analysis was performed using ISOQuant software v.1.7 [45,46]. The sequence length was set to a minimum of six amino acid residues with a minimum peptide score of six. The protein abundances were obtained using the TOP3-based quantification approach [45]. For comparative proteomics, after ISOQuant data analyses, only proteins present in all three biological replicates were considered for differential accumulation analysis, ensuring data quality. Proteins with significant differences according to Student’s *t*-test (two-tailed; *p* < 0.05) were considered to be differentially accumulated proteins (DAPs), which were considered to be up-accumulated if the Log_2_ fold change was greater than 0.6 and down-accumulated if the Log_2_ fold change was less than −0.6.

PCA was performed considering all proteins (missing values = 10) using prcomp::stats v.4.3.3 [47] and plotted using ggbiplot::ggbiplot v.0.55 [48] in the R language. The enrichment analysis of KEGG pathways and gene ontology terms (biological process, molecular function and cellular component) was performed using the IDs of the DAPs in g:Profiler with default settings for *Z. mays* [49]. A network of protein-protein interactions of the DAPs was constructed using the Search Tool for the Retrieval of Interacting Genes/Proteins (STRING) website [50], and a visualization was generated using the Cytoscape platform [51]. The protein–protein interaction network was built with orthologs of *Z. mays* proteins identified in *A. thaliana* using the STRING online tool (v 12.0). Redundant manual annotation was performed using the BLAST algorithm available on both the UniProtKB reference proteome plus SwissProt (https://UniProt.org/blast (accessed on 22 January 2024) and NCBI nonredundant protein sequences (https://blast.ncbi.nlm.nih.gov/Blast.cgi; accessed on 22 January 2024).

## 5. Conclusions

This study provides important insights into the molecular mechanisms underlying SLB resistance in popcorn by identifying DAPs associated with defense responses. The identification of specific metabolic pathways, such as photosynthesis, ribosome assembly, ascorbate and aldarate metabolism, glutathione metabolism, and carbon metabolism, suggests that these pathways are crucial in modulating the plant response to pathogen stress. Proteins such as PSB27-1 and RPP0B stand out as critical players in photosynthetic machinery and ribosomal protein synthesis, respectively, which are likely pivotal in sustaining both photosynthetic integrity and protein translation during SLB infection. Furthermore, the discovery of DHAR3 and its role in redox balance highlights its potential as a candidate for genetic improvement to increase resistance in popcorn varieties. Overall, this proteomic analysis offers a foundation for future research and breeding programs aimed at improving SLB resistance, paving the way for more resilient popcorn varieties in the face of increasing biotic stress challenges.

## Figures and Tables

**Table 1:** Differentially accumulated proteins exhibiting the same regulatory pattern in the SLB-resistant and SLB-susceptible genotypes comparisons at four (R4/S4) and ten (R10/S10) days after inoculation with *B. maydis*.

Accession	Arabidopsis Ortholog	Protein Description	KEGG-Enriched Metabolic Pathways	Differential Regulation *	
				R4/S4	R10/S10
B4F817	DHAR3	Dehydroascorbate reductase like3	Glutathione metabolism; Ascorbate and aldarate metabolism	UP	UP
B4FRJ4	PSB27-1	Photosystem II 11 kD protein	Photosynthesis	UP	UP
A0A1D6ETY3	CTIMC	Triose phosphate isomerase2	Carbon metabolism; Biosynthesis of amino acids; Biosynthesis of secondary metabolites; Carbon fixation in photosynthetic organisms; Glycolysis/gluconeogenesis	UP	Unique R10
A0A1D6FKV9	MMDH2	Malate dehydrogenase	Carbon metabolism; Biosynthesis of secondary metabolites; Carbon fixation in photosynthetic organisms; Citrate cycle (TCA cycle); Cysteine and methionine metabolism; Glyoxylate and dicarboxylate metabolism; Pyruvate metabolism	UP	Unique R10
A0A1D6LEZ7	RPP0B	60S acidic ribosomal protein P0	Ribosome	Unique R4	Unique R10
B6SID7	-	Late embryogenesis abundant protein, group 3	Not Enriched	Unique R4	Unique R10
B6UBR4	-	jacalin-like lectin	Not Enriched	Unique R4	Unique R10
B4FCH2	HTR4	Histone H3	Not Enriched	Unique R4	Unique R10
A0A1D6EH80	PETs	Elongation factor Ts, mitochondrial	Not Enriched	Unique R4	Unique R10
B4FVE8	VIPP1	Membrane-associated protein VIPP1 chloroplastic	Not Enriched	Unique R4	Unique R10
B6STA5	RBG7	Glycine-rich RNA-binding protein 2	Not Enriched	DOWN	DOWN
A0A1D6FUX8	SAM2	S-adenosylmethionine synthase	Biosynthesis of amino acids; Biosynthesis of secondary metabolites; Cysteine and methionine metabolism	DOWN	DOWN
A0A1D6EQY9	F4I18.32	Sterile alpha motif (SAM) domain-containing protein	Not Enriched	DOWN	DOWN
A0A1D6E9S6	F24G24.100	RmlC-like cupins superfamily protein	Not Enriched	DOWN	DOWN
B4FB80	TRXF2	Thioredoxin F2 chloroplastic	Not Enriched	DOWN	DOWN
K7TWV7	CYP19-1	Peptidyl-prolyl cis-trans isomerase	Not Enriched	DOWN	DOWN
K7UGF5	GAPC2	Glyceraldehyde-3-phosphate dehydrogenase	Carbon metabolism; Biosynthesis of amino acids; Biosynthesis of secondary metabolites; Carbon fixation in photosynthetic organisms; Glycolysis/gluconeogenesis	Unique S4	Unique S10
B4FRF0	GPX6	Glutathione peroxidase	Glutathione metabolism; Arachidonic acid metabolism	Unique S4	Unique S10

* R4 and R10: SLB-resistant genotype at 4 and 10 days after inoculation, respectively; S4 and S10: SLB-susceptible genotype at 4 and 10 days after inoculation, respectively.

## Data Availability

The mass spectrometry proteomics data and search results were deposited in the ProteomeXchange Consortium [52] by the PRIDE [53] partner repository with the dataset identifier PXD016768.

## Supplementary Materials

The following supporting information can be downloaded at: https://www.mdpi.com/article/10.3390/plants14030426/s1. Supplementary Table S1. Complete list of proteins identified in samples of popcorn maize leaves in a SLB-resistant genotype line at four days after inoculation with fungus *Bipolaris maydis* (R4), at ten days after inoculation (R10) and in a SLB-susceptible genotype at both times (S4 and S10). Supplementary Table S2. Gene ontology terms and KEGG pathways enriched among DAPs at four days after inoculation. Supplementary Table S3. Gene ontology terms and KEGG pathways enriched among DAPs at ten days after inoculation.
